# Exploring intentional medication non-adherence in patients with systemic lupus erythematosus: the role of physician–patient interactions

**DOI:** 10.1093/rap/rkaa078

**Published:** 2021-01-24

**Authors:** Jerik Leung, Elizabeth A Baker, Alfred H J Kim

**Affiliations:** 1 Department of Behavioral Science and Health Education, College for Public Health and Social Justice, Saint Louis University; 2 Division of Rheumatology, Department of Medicine, Washington University School of Medicine, Saint Louis, MO, USA

**Keywords:** systemic lupus erythematosus, qualitative research, medication adherence, social support, communication

## Abstract

**Objective:**

Medication non-adherence contributes to worse health outcomes among SLE patients. The underlying mechanisms that drive medication non-adherence are poorly understood. The purpose of this study was to explore possible mechanisms of medication non-adherence by eliciting patient experiences.

**Methods:**

Consented adult patients with ACR- or SLICC-classified SLE were recruited. Ten semi-structured interviews were conducted across six participants. Interviews were audio recorded, transcribed, and analysed using an iterative process. The findings were presented to an interactive public forum with SLE patients, family members and friends of patients, and health-care professionals to assess validity and for elaboration of the concepts developed.

**Results:**

The following three interrelated themes emerged from the interviews. First, why do rheumatologists not know more about lupus or share what they do know with their patients? Second, why do I have to take so many drugs and why do the drugs not work? Third, if my rheumatologist cannot communicate with me, why should I follow the prescribed medication regimen?

**Conclusion:**

Our exploratory findings lay out a possible underlying logic by which patients might choose intentionally to engage with medication non-adherence behaviours. Patients suggested that poor communication with their rheumatologists along with a lack of validation of their symptoms contributed to them not valuing the recommendations of physicians. This also contributed to development of a cynical outlook and little belief that medication would improve their condition. Although further work is needed to validate these findings, our preliminary work suggests that interventions focusing on the development of communication skills among both patients and rheumatologists are necessary to reduce medication non-adherence.

**Key messages**

- Poor communication between SLE patients and their rheumatologists is fundamental to medication non-adherence behaviours.
- Poor social support from the physician indirectly impacts health-related quality of life by increasing medication non-adherence.
- Interventions developing communication skills with rheumatologists and patients might improve physician– patient concordance, thereby reducing non-adherence.

## Introduction

SLE is a chronic autoimmune disease with a paroxysmal disease course [1]. SLE manifests in a wide variety of symptoms, such as joint pain, rash, photosensitivity and fatigue, and can also lead to irreversible organ damage, hospitalization or death [2].

SLE symptoms vary considerably across patients [3]. From the patient perspective, these symptoms vary in manifestations (i.e. what symptoms occur), timing (i.e. when and for how long certain symptoms manifest) and severity (i.e. how much impact do these symptoms have) [4]. The optimal treatment plan for each patient is individualized to a particular patient’s grouping of symptoms at a given time and is based on the best available information (e.g. laboratory values) in addition to past experiences of the clinician [3]. As a result, it is common for medication regimens to shift constantly in response to variation in patient symptoms. Most of the medication options available generally suppress immune system activity in order to alleviate symptoms, with a focus on minimizing disease activity. Additionally, many of the medications available have potential significant side effects, such as allergic reactions, cytopoenias, mood disorders, infertility, liver damage and risk of serious infections.

The constantly changing medication regimens and the wide variety of potential side effects present barriers to people taking their medication. This is often written about in the literature as a form of medical non-adherence. Medical non-adherence, defined as the level at which a patient’s medical behaviours do not align with the prescriber’s recommendations, is a persistent issue among individuals with SLE [5]. A recent review indicated that, in a majority of studies included on this topic, more than half of the study populations are medically non-adherent [6]. Non-adherence is an issue both with medications [7] and with other forms of clinical participation (e.g. individuals missing appointments) [8].

An additional consideration under medical non-adherence is intent. Medical non-adherence can be categorized as intentional or unintentional. The main distinguishing feature is whether there is a decision-making process associated with the medication non-adherence-related behaviour [9]. An example of intentional non-adherence might be deciding to not take a medication owing to a concern about negative side effects. An example of unintentional non-adherence might be an inability to obtain medications owing to cost. It is unclear whether one of these types of non-adherence, intentional or unintentional, is more prominent among SLE patients [9].

There is a significant amount of previous work examining medication non-adherence in SLE [6]. Although there are several issues limiting understanding and intervention, including an inconsistent definition of medication non-adherence and associated assessment instruments [10], it is clear that non-adherence is associated with increased health-care utilization [11] and worse outcomes [12, 13]. This is particularly concerning given previous qualitative work examining patient perceptions of SLE care [14] and quantitative, population-level work suggesting that SLE patients have consistently high non-adherence to their medication regimens [5].

Although medication non-adherence is associated with poor outcomes, the reasons remain unclear. By focusing on patient experiences and explanations for particular behaviours, qualitative work can assist in identifying these mechanisms, distinguishing pathways for intentional and unintentional non-adherence and suggesting potential strategies for intervention [15–18]. The purpose of this manuscript is to present findings from a qualitative study that explored challenges that patients experience in living with SLE, including medication adherence.

## Methods

### Study design

Using a phenomenological approach, we conducted semi-structured, qualitative interviews with SLE patients to gain a better understanding of their experiences of being diagnosed with and living with SLE [19]. Interviews took place in a variety of locations determined by the participants and provided both privacy for conversation and convenience for the study participant, including medical procedure rooms, libraries and individual homes. During some interviews, participants chose to bring a family member or supportive person. All interviews were conducted by J.L., a research team member who did not provide clinical care or have any prior relationship with the respondents. Two rounds of interviews were planned with study participants. The first interview was conducted using a semi-structured interview guide (Supplementary Data S1, available at *Rheumatology Advances in Practice* online) that consisted of questions and prompts to familiarize the researcher with the study participant, gain a sense of their path to diagnosis and discuss challenges they experience in living with SLE. The second round of interviews explored themes that emerged from the initial interviews further. Interviews averaged 50–70 min.

### Study participants and recruitment

Subjects with SLE classified using the ACR [20] or SLICC [21] classification criteria were recruited from the Washington University Lupus Clinic from June 2016 to August 2016 (demographic and clinical details of recruited subjects are provided in Table 1). We used a convenience sample, speaking to patients who came in for regularly scheduled appointments during the recruitment timeframe. No specific characteristics were sought. Of the 36 individuals to whom the project was introduced, 13 expressed interest in the project and consented to participate. All 13 of those individuals consented verbally at recruitment and were contacted for first-round interviews, six of which were scheduled and completed successfully. All 13 individuals were contacted twice to inquire about a first-round interview. Reasons for non-completion of interviews included unresponsiveness to interviewer messages and difficulties with scheduling. All six study participants were contacted for follow-up interviews after transcription and analysis of the initial interviews. Four follow-up interviews were scheduled and conducted successfully. A total of 10 interviews across 6 individuals were included for this analysis.

**Table 1:** Characteristics of respondents

Respondent	Race/ethnicity	Age (years)	Approximate time since diagnosis (years)	Interview round	S2K RI-50 score (closest visit to interview)	Medication (closest visit to interview)	FM (yes/no)
A	African-American	26	2	1	N/A	None	No
				N/A	N/A	None	
B	African-American	46	20	1	N/A	BEL HCQ	No
				2	N/A	BEL HCQ	
C	White	40	6	1	N/A	RTX MMF PDN	Yes
				N/A	N/A	N/A	
D	African-American	31	7	1	0	AZA HCQ PDN	No
				2	0	AZA HCQ PDN	
E	White	42	2	1	6	HCQ	No
				2	5	HCQ PDN	
F	White	40	7	1	0	LEU HCQ PDN	No
				2	0	HCQ PDN	

BEL: belimumab; PDN: prednisolone; HCQ: hydroxychloroquine; AZA: azathioprine; LEU: leflunomide; MMF: mycophenylate mofetil; RTX: rituximab; S2K RI-50: Systemic Lupus Erythematosus Disease Activity Index 2000 Responder Index-50

### Data analysis

Interviews were audiotaped, transcribed verbatim and reviewed for errors before beginning analysis. Open coding [22], whereby codes for recurring themes were generated as researchers read and re-read through the transcripts, was conducted. As analyses progressed, codes were revised and new codes added as appropriate. Transcripts were coded by an initial coder (J.L.); once all transcribed statements or quotes were assigned to a code, the codes and statements were printed and reviewed by two other members of the research team (E.A.B. and A.H.J.K.). This approach enhanced credibility of our data because it challenged the biases of the initial coder and enabled the coders to draw common conclusions supported by data. This step also allowed the researchers to determine whether quotes fitted within the assigned code or were better suited to others. All coding decisions were documented to provide an audit trail of the data analysis process. Inductive analysis was then used to identify themes [23]. During this step, codes were reviewed to identify groupings or themes and paragraphs drafted to summarize these themes in ways that remained grounded in the words and experiences of the patients.

During the qualitative analysis, we conceptualized data saturation as individually oriented, meaning that saturation was reached when concepts within individual interviews began to repeat, suggesting that we had reached a full understanding of the perspective of the respondent within the parameters of the interview [24].

These themes were presented, in a process known as member checking [25], to an interactive public forum of 40 participants including SLE patients, family members and friends of SLE patients, and health-care professionals. The analysis and themes were presented to the audience, and participants were asked to reflect and comment on these from their perspective. They were asked broad questions, such as the following: In what ways did the summary and themes represent your experiences? What would you add or take away from the themes as presented here?

Used in this way, member checking allowed for confirmation and/or disconfirmation of findings. It also provided a means for those interviewed and other individuals with SLE and their family members to provide additional information. This strengthens the validity of themes because it allows representatives of the population to evaluate the accuracy of themes directly [25, 26]. Although there were several broad themes emergent from the interviews and discussed during the member checking forum, this manuscript will focus only on themes related to medication and the patient–provider relationship, presented below with illustrative quotes.

### Ethics

This study was approved by the Washington University School of Medicine Institutional Review Board (protocol #201605104, initially approved 1 June 2016, last approved 26 June 2019).

## Results

Demographics of the clinical cohort are described in Table 1. All interview respondents identified as women; three individuals identified as African-American and three as White (Table 2). Respondents were between the ages of 26 and 46 years and had been diagnosed with SLE ≥2 years before the interview.

**Table 2:** Demographic and clinical characteristics of the clinical cohort from which researchers recruited study respondents

Characteristic	Value
Female, %	88
African-American, %	57.7
dsDNA-positive, %	67.8
Prednisone, %	46.8
Prednisone dose, mg, mean (s.d.)	16.14 (14.46)
S2K RI-50 score, mean (s.d.)	5.14 (5.74)
Age, years, mean (s.d.)	41.7 (13.3)
Blood iC3b, µg/ml, mean (range)	4.27 (0.7–21.0)
Blood iC3b/C3 ratio, µg/mg, mean (range)	4.49 (0.66–68.95)

Data are inclusive of 323 subjects with classified SLE. S2K RI-50: Systemic Lupus Erythematosus Disease Activity Index 2000 Responder Index-50; iC3b: iC3b is a protein generated with complement activation, a process that is central to the inflammatory nature of SLE; iC3b/C3 ratio: iC3b/C3 ratio is the proportion between iC3b levels and C3 levels. C3, when activated and broken down, generates iC3b.

Although these interviews were open ended and covered a wide range of topics, there was consistent focus of respondents on patient–provider interactions and experiences with medication. Three themes were identified from these responses. First, why do rheumatologists not know more about lupus or share what they do know with their patients? Second, why do I have to take so many drugs and why do the drugs not fully work? Third, if my rheumatologist cannot communicate with me, why should I follow the prescribed medication regimen?

### Why do rheumatologists not know more about lupus and share what they do know with their patients?

In general, many respondents indicated that they thought there was a gap between what their rheumatologist knew about SLE and the information she/he communicated with the respondent. In other words, many respondents did not always feel as if their rheumatologists were telling them the full story and perceived their rheumatologists as intentionally withholding information about SLE. Many respondents thought that more information from their rheumatologist would be better because it would give them some idea of what consequences to expect from their lupus (Table 3, Quote 1).

**Table 3:** Illustrative quotes: why do rheumatologists not know more about lupus and share what they do know with their patients?

These are things that doctors know … that it’s probably important for the patient to know, but they [rheumatologists] don’t necessarily think about telling the patient that. You don’t know how it’s [lupus] going to progress. They [rheumatologists] give you a spectrum … but they don’t know how it’s going to progress.… I think at least it would give you a heads up of what to expect [if more information was communicated to me]. Respondent F …And I’m like, how am I supposed to function after you take 15 vials of blood? I still to this day don’t know what it [all the blood work in a previous city] was for. They [current rheumatologist’s office] call, and they say, ‘Your lab results are fine’. They say ‘[rheumatologist] needs you to do this or that’. Nope, they don’t really tell you what they’re doing. Respondent A No, I don’t expect anybody [rheumatologists] will tell me anything.… I’m a fact-based person, you know … no one can deal with facts about anything [related to lupus]. And I understand and respect it [lack of concrete information]. At the same time … [it] is very frustrating. Respondent E This is my personal opinion, that people are so focused on their lane that they don’t see the overarching whole-body aspect of the cascade of symptoms and how it’s all interrelated. And how all the systems are working together to kind of set you up unless you manifest with these specific symptoms and we can call them lupus. But that no one really understands the overall mechanism. Respondent E

This sense of intentional withholding of information persisted when some respondents spoke about their interactions with rheumatologists with regard to laboratory/clinical tests. Some respondents noted that they did not fully understand the purpose of some laboratory tests or clinical work, particularly blood work. They attributed this poor understanding directly to instances in which their rheumatologist did not always tell them what type of test was performed and the reasoning behind particular tests or laboratory work (Table 3, Quote 2). This lack of shared information was seen as contributing to poor understanding of both general and laboratory/clinical work specific to SLE. This also contributed to an assumption, and at times an expectation, that their physicians might not be telling them anything about their condition because even the physicians have minimal knowledge of the ‘facts’ about lupus (Table 3, Quote 3).

Another point of frustration among some respondents was the narrow focus of many specialized physicians, such as rheumatologists. Several respondents perceived that their rheumatologists do not understand the overall mechanisms of autoimmune diseases and are so focused on lupus that they have a hard time addressing the cascade of symptoms that many lupus patients have outside of what their rheumatologist defines as lupus related (Table 3, Quote 4).

### Why do I have to take so many drugs and why do the drugs not fully work?

The perception that rheumatologists do not understand enough about lupus and do not communicate what they do know to their patients led many respondents to question the medication regimen they were prescribed. Several respondents stated that rheumatologists were not able to communicate the purpose or logic behind prescribing certain medications. This was evidenced by rheumatologists telling patients to take medication but being unwilling or unable to explain why respondents should take the medication if they felt better when not taking their medication (Table 4, Quote 1).

**Table 4:** Illustrative quotes: why do I have to take so many drugs and why do the drugs not work?

…[My rheumatologist] got mad because I stopped taking my medicine. I was like, if I feel better when I’m off this pill than when I’m on the pill then why should I take it? [My rheumatologist] said, ‘Well we’re trying to control for kidney damage. You’re too young for kidney damage.’ Again, [I said] if I felt better off this pill than on this pill, then why would I continue to take it? [My rheumatologist just says again,] ‘You’re not following the treatment.’ Respondent A I feel tested on. I have been on so many medications.… One time I was taking over 13 pills a day. You feel horrible after putting all these things in your body.… Respondent B All of that medicine together … as much as they [rheumatologists] tell you that it’s [medication] gonna help you, it doesn’t.… Respondent A It [medication] helps with my weight gain. And maybe a few less flare ups. But I still have them. It [medication] may lessen it [pain, symptoms] but it doesn’t eliminate them. I’m still on the same drugs with the [belimumab]. I’ve been on steroids for 16 years, I would lose weight. I would have problems with weight loss. I would go 3 days without eating anything. Respondent B Belimumab used to be my best friend but it [positive effects] didn’t last long. Lupus requires a lot of medicine because it starts to bother different things. The numbing and tingling stuff in my fingers and toes.… Respondent D Yeah, another side effect that I didn’t know.… The whole side of my face will go numb. I can feel it when it’s coming too. It just looks like really … scary. Like I’m about to have a stroke or something. Respondent B [Mother of respondent speaking] Some days it’s bad. Some days is good. Her [SLE patient] joints swell up. They really, really bad. She has short-term [memory loss]. She don’t [sic] sleep at night. She up and down all night. Then she start [sic] taking the, uh, infusions. She had gained a little weight, and she eat more. Because she never have [sic] the appetite to want more food. But now that she start on the infusions, it seems to be helping for the weight. Sometimes think, I know it’s [the infusion] helping her, but I’m like … is it [infusions] worth it? Is she ever able to get off of it? Or do you have to come and do this [infusions] the rest of your life? I don’t know. Respondent B

Although some respondents recognized some of the positive benefits of the medication, they disliked the sheer quantity of different medications they had to take. In some cases, respondents indicated that the variety and number of medications prescribed left them feeling ‘tested on’ by their rheumatologists (Table 4, Quote 2).

The concerns of several respondents went beyond the simple number of pills they need to take. They raised concerns that their rheumatologist was not able to help them set realistic expectations. They remarked that their rheumatologist said that their medication would help their lupus symptoms but that they became frustrated when that expectation was not met (Table 4, Quote 3).

Some respondents were also frustrated that their medication affected only certain symptoms (not all symptoms) and that even those symptoms were only lessened, not eliminated (Table 4, Quote 4).

Another concern raised was that the positive effects of each medication were not maintained. As a result, there was an overarching frustration of being trapped in a cycle of always looking for the next thing that might help (Table 4, Quote 5).

A few respondents also spoke of concerns about side effects, emphasizing that these side effects were scary and that their unpredictability did not allow respondents to know what to expect (Table 4, Quote 6). Some respondents subsequently began to question whether the supposed benefits of the medication were worth these side effects and the toll that their medication had on themselves and their family (Table 4, Quote 7).

### If my rheumatologist cannot communicate with me, why should I follow the prescribed medication regimen?

When describing their communication with their rheumatologists, many respondents not only spoke about feeling that their physicians were not providing sufficient information but also often spoke about a frustration with feeling that their rheumatologist was not listening to them and wishing that their rheumatologist was more willing to acknowledge their experiences and their specific lupus symptoms. They felt that their rheumatologist talked ‘at them and not to them’ (Table 5, Quote 1).

**Table 5:** Illustrative quotes: if my rheumatologist cannot communicate with me, why should I follow the medication regimen they prescribe?

So, just because it’s something that you know or something what the book says, doesn’t mean that it’s me. It would be better if [my rheumatologist] listen and take everything on a case by case. Instead of just putting me in the lupus pile.… But [my rheumatologist] talks at me and not to me … It’s a counter-reaction to whatever I said.… Respondent B This [infusion] feels cold. With the lupus, because I have the [RP]. I have my little jacket to protect from the UV rays. This changes my body temperature. When it [infusion] goes in, it feels like winter. Yeah, that’s my life. I don’t know if it’s working. Do I have to do this for the rest of my life? Do I get any time off? They [rheumatologists] study it [lupus] but they don’t know. Respondent B They try and tell me how I feel. Or, ‘Oh no, the drugs don’t do that’. It does it to me. You [rheumatologist] don’t take the drug; I take the drug. Sometimes, I shut down. And after I tell you how I feel, and you tell me that’s wrong, I get frustrated. You not listening. Just because you did this [treatment] for somebody else does not mean it will work same way in me.… It [drug] may do this for them [other patient] but it doesn’t do this for me. Respondent B So, my theory behind my medication is, if I’m feeling good today, then I’m not taking my medication today. Respondent D I take it [medication] now so I don’t have to fight [my rheumatologist].… I pick and choose. I pick and choose. There will be days where I’ll be like, whatever, I’m not taking it.… Respondent A I don’t care if people diagnose [me] with jack shit anymore. I just go to the doctor and make sure my junk isn't blowing up anywhere … to make sure I don't die before I raise my children. I don't even care what it's [lupus] called, quite frankly.… I have no expectation that anyone can tell me what is causing what. I think that for the most part, the medical community, they kind of know some stuff and the rest is a crapshoot of guessing. Respondent E

A few respondents also voiced concerns that rheumatologists do not really understand the perspective or experience of someone who has to take the SLE medications. For instance, one respondent highlighted this by distinguishing between simply ‘studying’ lupus (i.e. rheumatologist perspective) and really ‘knowing’ lupus (i.e. patient perspective) (Table 5, Quote 2).

A specific manifestation of these communication challenges was not feeling validated by their rheumatologist when it came to describing how a particular medication or grouping of medications might be affecting them. Some respondents were frustrated when rheumatologists told them how a medication should be affecting them rather than listening to and acknowledging respondents’ explanations of how a medication was really affecting them. As a result, some respondents indicated that they sometimes ‘shut down’ at the rheumatologist’s office (Table 5, Quote 3).

For a few respondents, one consequence of the inability to have meaningful communication with their rheumatologist about their condition and treatment of it was intentionally altering their medication regimen. As one respondent said, they might choose to not take their medication if they’re feeling good on a particular day (Table 5, Quote 4). Notably, several respondents indicated that they might take their medications in order to avoid fighting with their rheumatologist but still might have a ‘pick and choose’ strategy, deciding not to take their medications on a given day (Table 5, Quote 5).

As a result, many respondents portrayed a cynical attitude towards the prospect of their illness. They had little expectation that physician management would improve their condition, meaning that the best-case scenario was to focus on avoiding catastrophe (Table 5, Quote 6).

## Discussion

We have laid out a patient perspective of an underlying logic by which individuals might intentionally choose to modify or disengage from their SLE medication regimen. Our exploratory data suggest that a root cause for this behaviour is poor interactions with rheumatologists. Respondents observed a gap between what they think their rheumatologists know about their patients’ SLE treatment (e.g. laboratory/clinical tests, potential side effects of medication) and what their rheumatologists communicate to them. These interactions contributed to the frustration of respondents and made them feel that they did not fully understand the purpose of their medications. When respondents did try to communicate to their physician about their SLE and how medication might be affecting them, they often felt invalidated when the side effects or symptoms they experienced did not align with what was expected from the physician. This then formed a basis for development of distrust between respondents and their rheumatologists, which in turn led to respondents doubting the effectiveness of their prescribed medication. As a result, respondents expressed a cynical attitude towards the potential gain of taking medication, describing strategies for intentionally altering or not taking medications.

Our data portray poor physician–patient communication as a root cause of medication non-adherence. A key point suggested by respondents was that they often felt invalidated by the way that their rheumatologist communicated with them, which suggests an overall lack of empathy and unwillingness or inability of rheumatologists to understand the patient experience. This feeling of invalidation contributed to the respondents’ distrust of their rheumatologists.

This portrayal of poor communication between rheumatologists and patients as a root cause of medication non-adherence in SLE patients aligns with other findings regarding medication non-adherence among SLE patients. Although there are difficulties with measuring medication non-adherence, recent reviews have indicated that determinants of medication non-adherence include both polypharmacy [6, 27, 28] and the quality of the doctor–patient relationship [5]. However, our data add depth to this potential root cause of intentional medication non-adherence by providing a logical thread explaining patient reasoning. The open-ended nature of our interviews suggests that this intentional decision to change medication regimens is core to the patient experience with SLE.

In addition, although previous studies have typically identified the quality of the doctor–patient relationship as a determinant of non-adherence, those studies have not identified a potential pathway. Our data indicate that poor communication and not feeling validated by their rheumatologists are ways in which SLE patients operationalize social support, specifically informational support (poor communication) and appraisal support (lack of validation) [29]. Deficiencies in these two types of social support are thought to impact health-related quality of life directly. What our data suggest is that these aspects of social support might also have an indirect effect on health-related quality of life by impacting medication non-adherence [30–32].

Developing methods of reducing communication obstacles between patients and their rheumatologists is crucial. One barrier to this might be the general framing implied by the word adherence. Although adherence itself is seen as movement away from the more paternalistic word compliance, some see adherence as still placing the onus on the patient to follow treatment recommendations, even if those recommendations are set cooperatively by the physician and the patient. Moving towards a standard of concordance [33], which implies a shared responsibility in coming to medical decisions and, more appropriately, considers communication as a bi-directional process, might be important to consider for future communication interventions.

### Limitations

The exploratory nature and the small sample size limit broad generalizability of our findings. The goal within a phenomenological approach is to ensure in-depth understanding, and that understanding potentially leads to new lines of research. Thus, although our small sample size (10 interviews across 6 individuals, with 40 audience members during the interactive forum) limits the broad applicability of these findings to SLE at a population level, our second interviews with respondents and member checking facilitated a full and complete accounting of the perspective of each respondent. In addition, our conceptualization of saturation was individually oriented rather than across the entire sample [24, 34]. A limitation of this orientation is that there could be topics relevant to the patient experience, in addition to the ones presented here, that might be relevant. For instance, racial and cultural differences between the patient and the provider have been noted as a factor relevant to patient–provider interactions in medication adherence [35], but this was not a theme in our interviews.

An additional limitation is the lack of opposing viewpoints on medication from our sample. This lack of inclusion could be a source of bias because our sample might reflect those individuals who were dissatisfied with their treatment.

A further limitation is that socioeconomic factors and race were not addressed explicitly during the data collection or analysis. The racial and gender variability in our sample was limited (all female, only White or African-American), owing, in party, to the demographic characteristics of the available cohort (Table 2). Although there was not a male perspective of SLE in the interview portion of this qualitative work, we made efforts to include men and introduced the project to several male patients. The difficulty of recruiting male participants was compounded by their low representation in the available clinical cohort (Table 2), which is a consistent finding in human subject research in SLE [36]. Future studies would need to account better for the demographic variations, particularly those examining larger populations, because these individuals with differing racial and gender identities might offer additional perspectives/experiences of SLE.

### Future research

Although further work is needed to investigate fully the reasoning outlined here for intentional non-adherence, there are several implications for future studies and interventions. There is a need to develop specific intervention activities related to improving communication that address both patient and physician barriers sufficiently and equitably. Our work suggests that the primary focus on the patient side should be in developing patient skills and confidence in preparing for their physician appointments. Specific considerations for patients with SLE will need to include methods of symptom documentation, asking clarifying questions and adjusting expectation setting for future treatments.

There are also possible suggestions for the physician side. For example, physicians might need additional training on how to balance the need for communicating with patients in ways that enhance physician–patient understanding with the increasing time spent on electronic medical records and pressures to see a high volume of patients [37, 38]. This tension might be particularly challenging for rheumatologists who focus on autoimmune diseases, such as SLE, where there might be a greater patient need for both information and validation (i.e. appraisal support) related to SLE-specific experiences, especially given the impact of these types of support on SLE patient health-related quality of life [29]. Among rheumatologists who treat SLE, there are ongoing efforts to change norms of discussing SLE symptoms by introducing a categorization scheme which might aid in validating patient experiences [39, 40].

An additional barrier for physician communication specifically is the lack of archetypal patients. There is not necessarily a book definition of SLE symptoms or approach to diagnoses. This ambiguity poses a unique barrier to physicians who encounter SLE patients and might require development of specific communication skills for physicians on how to discuss this ambiguity.

In addition, there are structural considerations related to physician communication. Although skills such as intentional listening might by emphasized in early medical school education, there might be some level of attrition [41] when moving into graduate-level medical training as new physicians begin to develop routines and expectations for the patient interview and might have a lesser emphasis on listening to patient concerns. This means that communication skills interventions might need to be dynamic and routine rather than static and single time.

## Supplementary Material

rkaa078_Supplementary_Data
