# Supplementary material for: Exploring intentional medication non-adherence in patients with systemic lupus erythematosus: the role of physician–patient interactions
Source: Rheumatol Adv Pract. 2021 Jan 24;5(1):rkaa078. doi: 10.1093/rap/rkaa078 (PMC7878846; doi:10.1093/rap/rkaa078)
Supplement: rkaa078_Supplementary_Data [file rkaa078_supplementary_data.pdf]

Supplementary Data S1. Interview guide

1.) Do you have any questions for me about the purpose or goals of this study?

2.) How would you define Lupus?

3.) What is unique to Lupus compared to other illnesses?

4.) How long did it take for you to become diagnosed with Lupus?

  a. How many doctors did you see during that time?

5.) What were the obstacles in the diagnosis process?

6.) How has Lupus affected your everyday life?

7.) How has Lupus affected your family life?

8.) How has Lupus affected your social relationships?

9.) How has Lupus affected your employment?

10.) How do you think other people perceive Lupus?

11.) Do you know other people with Lupus?

12.) Discussion Topic: Obstacles that you have encountered related to your illness
